# Serum uric acid is associated with coronary artery calcification in early chronic kidney disease: a cross-sectional study

**DOI:** 10.1186/s12882-021-02463-2

**Published:** 2021-07-04

**Authors:** Miyeun Han, Hyunsuk Kim, Hyo Jin Kim, Eunjeong Kang, Yong-Soo Kim, Kyu Hun Choi, Soo Wan Kim, Curie Ahn, Kook-Hwan Oh

**Affiliations:** 1 Department of Internal Medicine, Hallym University Hangang Sacred Heart Hospital, Seoul, Korea; 2 Department of Internal Medicine, Hallym University Chuncheon Sacred Heart Hospital, Chuncheon, Korea; 3 Department of Internal Medicine, Pusan National University Hospital, Busan, Korea; 4 Department of Internal Medicine, Ewha Womans University Seoul Hospital, Ewha Womans University College of Medicine, Seoul, Korea; 5 Department of Internal Medicine, The Catholic University of Korea, Seoul St. Mary’s Hospital, Seoul, Korea; 6 Department of Internal Medicine, College of Medicine, Institute of Kidney Disease Research, Yonsei University, Seoul, Republic of Korea; 7 Department of Internal Medicine, Chonnam National University Medical School, Gwangju, Korea; 8 Division of Nephrology, Department of Internal Medicine, Seoul National University College of Medicine, 101 Daehak-ro, Chong No Gu, 03080 Seoul, Korea

**Keywords:** Uric acid, Chronic kidney disease, Coronary artery calcification, Coronary computed tomography

## Abstract

**Background:**

Although uric acid (UA) is regarded as a risk factor for cardiovascular disease, whether UA is an independent risk factor contributing to coronary artery calcification in chronic kidney disease (CKD) is not well known. We evaluated whether UA level is associated with coronary artery calcium (CAC) score in a predialysis CKD cohort.

**Methods:**

A total of 1,350 subjects who underwent coronary computed tomography as part of the KoreaN Cohort Study for Outcome in Patients With Chronic Kidney Disease were analysed. We conducted a logistic regression analysis to evaluate the association between UA and the presence of CAC.

**Results:**

CAC was detected in 705 (52.2 %) patients, and the level of UA was significantly higher in CAC > 0 patients. UA showed a positive relationship with CAC > 0 in age- and sex-adjusted logistic regression analysis (Odds ratio (OR) 1.11, 95 % confidence interval (CI) 1.04–1.19, *P* = 0.003). However, UA showed no association with CAC > 0 in multivariate analysis. Further analysis showed that UA showed a positive association with CAC > 0 only in estimated glomerual filtration rate (eGFR) > 60 ml/min/1.73 m^2^ (OR 1.23, 95 % CI 1.02–1.49, *P* = 0.036) but not in eGFR 30–59 ml/min/1.73 m^2^ (OR 0.92, 95 % CI 0.78–1.08, *P* = 0.309) or < 30 ml/min/1.73 m^2^ (OR 0.92, 95 % CI 0.79–1.08, *P* = 0.426).

**Conclusions:**

UA level was significantly associated with CAC in early CKD, but not in advanced CKD.

**Supplementary Information:**

The online version contains supplementary material available at 10.1186/s12882-021-02463-2.

## Background

Cardiovascular disease (CVD) is the leading cause of morbidity and mortality in patients with chronic kidney disease (CKD), accounting for more than half of the deaths [1]. Patients with CKD have traditional risk factors for CVD, such as hypertension, diabetes, dyslipidemia, and elderly. In addition, CKD-related risk factors such as endothelial dysfunction, hyperphosphatemia, vascular calcifications, and chronic inflammation play important roles in the advancement of the disease [2]. Vascular calcification is a common complication in CKD, resulting from altered mineral homeostasis and an imbalance of calcification promoters and inhibitors [3, 4]. The extent and anatomic type of calcification are well-known predictors of vascular mortality [5].

Coronary artery calcification is significantly associated with the occurrence of major cardiovascular events. As measured by multi-detector computed tomography, the degree of calcification is one of the most widely accepted tools for detecting coronary atherosclerosis and estimating coronary risk assessment with high sensitivity and specificity [6]. This tool is valid not only in asymptomatic adults [7, 8] but also in the CKD population [9]. Therefore, the coronary artery calcium (CAC) score is now used to assess cardiovascular risk in patients with CKD.

Uric acid (UA), the end product of purine catabolism, has been in the spotlight for decades as a risk factor for cardiovascular disease. Many epidemiologic studies have revealed that hyperuricemia is associated with an increased risk of coronary heart disease [10], heart failure [11], and fatal arrhythmias [12]. UA showed a positive association with CAC in health screening for the general population [13–16] and elevated UA could predict the development of coronary artery calcification progression [17]. However, few studies have investigated the relationship between UA and CAC in the CKD population. Therefore, in this study, we sought to demonstrate whether serum UA level is associated with CAC in the predialysis CKD population.

## Methods

### Study design and population

This was a cross-sectional study designed to assess the association between serum UA and CAC in patients with CKD. The study population consisted of participants in the KoreaN Cohort Study for Outcome in Patients With Chronic Kidney Disease (KNOW-CKD), a nationwide prospective cohort study, including CKD stage 1 to 5 nondialysis patients, that was designed to clarify the natural course and complication profiles of CKD. Details of the KNOW-CKD study’s rationale and design have been described previously [18] and the baseline characteristics of the study population were previously published [19]. A total of 2,238 subjects were initially screened. After excluding 126 participants who had not performed coronary artery computed tomography, 434 participants who had taken UA lowering medication, and 328 participants with missing information in any one of the variables included in the study, 1350 participants were analysed.

### Data collection

Baseline demographics and laboratory data were retrieved from the electronic data management system (PhactaX, Seoul, Republic of Korea) with assistance from the Division of Data Management at Seoul National University Medical Research Collaborating Center. Socio-demographic characteristics, smoking history, medication, and comorbidities were collected at the time of enrollment. Anthropometric indexes including height, weight, waist circumference, hip circumference, and resting blood pressure (BP) were measured in the clinic. Laboratory data were analysed at the hospital laboratory of each participating centre. Serum samples were collected and sent to the central laboratory (Lab Genomics, Seoul, Republic of Korea) to measure creatinine levels. The estimated glomerular filtration rate (eGFR) was calculated through the CKD-EPI equation [20].

CAC score was evaluated at each research institution by an electron-beam computed tomography (CT) scanner or a multi-detector CT. Specifically, SOMATOM Definition Flash (Siemens Healthineers), SOMATOM Force (Siemens Healthineers), Revolution CT (GE healthcare), Discovery CT750 HD (GE Healthcare), Brilliance iCT 256 slice (Philips Medical Systems), Brilliance 64 CT scanner (Philips Medical Systems), IQon Spectral CT (Philips Medical Systems) were used. The quantitative CAC score was assessed using the Agatston score [21]; If a lesion with a density of 130 Hounsfield unit (less than HU) and a width of 1 mm^2^ or more is in the coronary artery position, it is determined as positive. In each lesion, 1 point for 130 to 199 HU, 2 for 200 to 299 HU Points, 3 points for 300 ~ 399 HU, 4 points for 400 or more, were measured as the sum of these values.

### Statistical analysis

Continuous variables were expressed as mean ± standard deviation, and categorical variables were expressed as numbers and proportions. The comparisons were made using the independent t-test or analysis of variance for continuous variables and the chi-squared test for categorical variables. A *P*-value < 0.05 was considered statistically significant. The adjusted odds ratios of CAC > 0 associated with risk factors were estimated using logistic regression analysis. Only covariates that were significant (*p* < 0.05) were retained in the multivariable analysis. The independent association between UA and CAC was also evaluated using restricted cubic splines. To visualise the association of UA and CAC, selected knots automatically, cubic splines were drawn by the “gam,” “mgcv” packages in R. Statistical analysis was performed using R software (version 3.2.2).

### Ethics statement

This study was conducted in accordance with the Declaration of Helsinki. The study protocol was approved by the institutional review board at each participating hospital, including Seoul National University Hospital (1104-089-359), Seoul National University Bundang Hospital (B-1106/129-008), Yonsei University Severance Hospital (4-2011-0163), Kangbuk Samsung Medical Center (2011-01-076), Seoul St. Mary’s Hospital (KC11OIMI0441), Gil Hospital (GIRBA2553), Eulji General Hospital (201105-01), Chonnam National University Hospital (CNUH-2011-092), and Pusan Paik Hospital (11–091). All participants provided written informed consent.

## Results

### Clinical characteristics of participants with CAC

The mean age was 53.5 ± 11.9 years, and 758 (56.2 %) were men. Seventy (5.2 %) had a history of coronary artery disease, 499 (37.0 %) had diabetes mellitus, and 1286 (95.3 %) had hypertension. The mean eGFR was 56.7 ± 32.4 ml/min/1.73 m^2^, and the serum UA level was 7.0 ± 1.9 mg/dL. Of these, 645 (47.8 %) belonged to CACS 0 and 705 (52.2 %) belonged to CACS > 0. Table 1 shows the clinical characteristics of CAC 0 and CAC > 0. Compared to those with CAC 0, participants with CAC > 0 were older and had a higher proportion of males. Comorbidities, such as coronary artery disease, diabetes mellitus, and hypertension, were higher in CAC > 0, and the proportion of current smokers and lipid-lowering medications was also higher. The UA, phosphate, PTH, and 24-hour urine protein levels were significantly higher in participants with CAC > 0. In contrast, the levels of eGFR, calcium, LDL-cholesterol, and HDL-cholesterol were significantly lower in CAC > 0.

**Table 1:** Clinical characteristics of participants

	Total (*n* = 1350)	CAC 0 (*n* = 645)	CAC > 0 (*n* = 705)	*P* value
Age, year	53.5 ± 11.9	47.6 ± 11.3	58.8 ± 9.8	< 0.0001
Sex, male (%)	758 (56.2)	277 (43.0)	481 (68.2)	< 0.0001
Coronary artery disease (%)	70 (5.2)	2 (0.3)	68 (9.7)	< 0.0001
Diabetes mellitus (%)	499 (37.0)	97 (15.0)	402 (57.0)	< 0.0001
Hypertension (%)	1286 (95.3)	593 (91.9)	693 (98.3)	< 0.0001
current smoker (%)	217 (16.1)	88 (13.6)	129 (18.3)	0.020
Use of lipid-lowering drugs (%)	727 (53.9)	279 (43.3)	448 (63.6)	< 0.0001
Mean blood pressure, mmHg	94.0 ± 11.6	93.1 ± 11.3	94.8 ± 11.9	0.005
Waist hip ratio (%)	89.8 ± 7.2	87.6 ± 7.8	91.8 ± 5.8	< 0.0001
Creatinine, mg/dL	1.72 ± 1.12	1.44 ± 0.93	1.98 ± 1.21	< 0.0001
eGFR, ml/min/1.73m^2^	56.7 ± 32.4	67.3 ± 33.9	47.1 ± 27.5	< 0.0001
Uric acid, mg/dL	7.0 ± 1.9	6.7 ± 2.0	7.3 ± 1.9	< 0.0001
Calcium, mg/dL	9.1 ± 0.5	9.2 ± 0.49	9.1 ± 0.6	< 0.0001
Phosphate, mg/dL	3.7 ± 0.7	3.6 ± 0.6	3.8 ± 0.7	< 0.0001
LDL cholesterol, mmol/L	98.4 ± 31.9	102.6 ± 29.9	94.5 ± 33.1	< 0.0001
HDL cholesterol, mmol/L	50.0 ± 15.8	53.6 ± 15.5	46.8 ± 15.4	< 0.0001
Ln PTH, pg/mL	3.95 ± 0.74	3.85 ± 0.70	4.05 ± 0.77	< 0.0001
Ln 24 h urine protein, mg	6.1 ± 1.8	5.7 ± 1.9	6.5 ± 1.6	< 0.0001

*eGFR* estimated glomerular filtration rate, *LDL* low density lipoprotein, *HDL* high density lipoprotein, *PTH* parathyroid hormone

We further divided as CAC 0, > 0–100, > 100–400, > 400. The participants with high CAC score were older and had higher proportion of male, coronary artery disease, diabetes mellitus and hypertension. The level of eGFR, calcium, LDL cholesterol, and HDL cholesterol were lower, whereas uric acid, phosphate, PTH, and 24 h urine protein were higher according to higher CAC level (Supplementary Table 1).

### Association factors with CAC

Table 2 lists several factors associated with CAC > 0. The age- and sex-adjusted logistic regression analysis revealed UA (Odds ratio (OR) 1.11, 95 % confidence interval (CI) 1.04–1.19, *P* = 0.003) was significantly associated with having CAC > 0. However, on multivariate logistic regression analysis, adjusted for age, sex, coronary artery disease, diabetes mellitus, use of lipid-lowering agents, mean BP, waist-hip ratio, eGFR, calcium, phosphate, LDL-cholesterol, HDL-cholesterol, PTH, and 24-hour urine protein, UA showed no association with CAC > 0 (OR 1.00, 95 % CI 0.91–1.09, *P* = 0.953). History of coronary artery disease, history of diabetes mellitus, use of lipid-lowering agents, mean BP, and phosphate levels were positively associated with CAC in multivariate analysis.

**Table 2:** Multivariate-adjusted odds ratios of CAC >0 associated with several risk factors

	age-, sex- adjusted OR		Multivariate-Adjusted OR	
	OR (95% CI)	*p*-value	OR (95% CI)	*p*-value
Current smoker	1.29 (0.90, 1.85)	0.169	-	-
Coronary artery disease	23.0 (5.3, 100.6)	<.0001	10.44 (2.45, 44.48)	0.002
Hypertension	2.04 (0.98, 4.24)	0.057	-	-
Diabetes mellitus	5.12 (3.83, 6.86)	<.0001	3.53 (2.56, 4.87)	<.0001
Use of lipid-lowering drugs	1.66 (1.29, 2.14)	<.0001	1.37 (1.02, 1.84)	0.039
Mean blood pressure	1.02 (1.01, 1.03)	<.0001	1.02 (1.01. 1.03)	0.006
Waist hip ratio	1.05 (1.03, 1.07)	<.0001	1.02 (1.00, 1.05)	0.075
eGFR	0.99 (0.98, 0.99)	<.0001	1.00(0.99, 1.01)	0.591
Uric acid	1.11 (1.04, 1.19)	0.003	1.00 (0.91, 1.09)	0.953
Calcium	0.64 (0.50, 0.81)	<.0001	0.83 (0.61, 1.11)	0.206
Phosphate	1.82 (1.47, 2.24)	<.0001	1.31 (1.02, 1.68)	0.032
LDL cholesterol	1.00 (0.99, 1.00)	0.038	1.00 (1.00, 1.00)	0.775
HDL cholesterol	0.98 (0.97, 0.99)	<.0001	0.99 (0.98, 1.00)	0.171
Ln PTH	1.49 (1.25, 1.78)	<.0001	1.03 (0.81. 1.32)	0.793
Ln 24hour urine protein	1.24 (1.14, 1.34)	<.0001	0.99 (0.90, 1.10)	0.909

*eGFR* estimated glomerular filtration rate, *LDL* low density lipoprotein, *HDL* high density lipoprotein, *PTH* parathyroid hormone

**Fig. 1:**
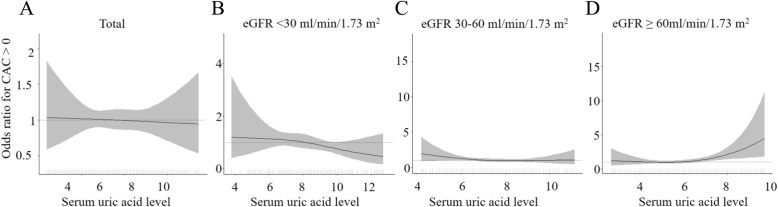
Restricted cubic splines of serum uric acid on the coronary artery calcium score > 0, stratified by CKD stage. **A** total, **B** eGFR < 30 ml/min/1.73m^2^, **C** eGFR 30–59 ml/min/1.73m , **D** eGFR ≥ 60 ml/min/1.73m^2^

We further divided the participants by eGFR ≥ 60, 30–59, and < 30 ml/min/1.73 m^2^. UA level showed a positive association with CAC > 0 (OR 1.23, 95 % CI 1.02–1.49, *P* = 0.036) only in estimated GFR > 60 ml/min/1.73 m^2^ (Table 3). UA had no association with CACS in eGFR 30–59 ml/min/1.73 m^2^ (OR 0.92, 95 % CI 0.78–1.08, *P* = 0.309) or 30 ml/min/1.73 m^2^ (OR 0.92, 95 % CI 0.79–1.08, *P* = 0.426). Restricted cubic splines of UA on the CAC > 0, stratified by eGFR, showed that CAC increased steadily with higher UA levels only in estimated GFR > 60 ml/min/1.73 m^2^ (Fig. 1).

**Table 3:** Multivariate-adjusted odds ratios (95 % Confidence Intervals) for CAC > 0 associated with serum uric acid according to CKD stages

	n	OR (95 % CI)	*p*-value
eGFR ≥ 60ml/min/1.73 m^2^	541		
Uric acid		1.23 (1.02, 1.49)	0.036
eGFR 30-59ml/min/1.73 m^2^	475		
Uric acid		0.92 (0.78, 1.08)	0.309
eGFR < 30 ml/min/1.73 m^2^	334		
Uric acid		0.92 (0.79, 1.08)	0.426

*CKD* chronic kidney disease

## Discussion

In the present study, we showed that over half of the CKD population had CAC. The patients with CAC > 0 had higher UA levels than those with CAC 0, and UA showed a positive relationship with CAC > 0 in age- and sex-adjusted logistic regression analysis. However, UA showed an association with CAC > 0 in multivariate-adjusted logistic regression analysis only in the early stage of CKD, and this association disappeared in advanced CKD.

UA, formerly considered a significant antioxidant in humans, has been regarded as a risk factor for cardiovascular disease in the past two decades. Previous studies have shown that elevated UA levels could predict the severity and morphology of coronary atherosclerosis [16] and elevated UA is associated with greater lipid content of coronary plaque in patients with acute coronary syndrome [22]. In line with this, hyperuricemia showed a positive association with CAC in the general population in previous studies [13, 15]. Several experimental studies have demonstrated the mechanism of UA in atherosclerosis. In endothelial cells, UA decreases nitric oxide bioavailability and inhibits cell migration and proliferation [23, 24]. UA significantly increased the production of reactive oxygen species, facilitated the activation of the renin-angiotensin system [25], and induced endothelial dysfunction. In addition, UA has been shown to have pro-inflammatory activity. UA triggers the expression of pro-inflammatory cytokines such as C-reactive protein [26, 27], contributing to atherosclerosis.

However, UA failed to show an association with CAC in patients with CKD, not only in this study population but also in another CKD cohort [28]. The authors believe that this is due to the complex process of vascular calcification in CKD. Vascular calcification can occur in two areas of the vessel wall: the intima and the media. Intima calcification is associated with atherosclerosis, whereas calcification in the media is associated with advanced age, diabetes, and CKD [29]. Abnormal calcium and phosphate levels and parathyroid hormone levels in kidney disease contribute to the phenotype switch of vascular smooth muscle cells to osteoblast-like cells and local inflammation, contributing to vascular calcification [4].

Unlike UA, phosphate showed a positive association with CAC throughout the whole stage of CKD in this study. Phosphate is an integral component of hydroxyapatite, the calcium mineral seen in bone and vascular calcification. It is also a part of the signalling cascade that triggers vascular calcification [29]. Calcification of the coronary arteries is independently associated with hyperphosphatemia in patients with end-stage renal disease, and elevated phosphate levels, even those in the high-normal range, are correlated with a higher risk of myocardial infarction and mortality in patients with CKD [30].

This study has some limitations. This is a cross-sectional study; therefore, we could not identify a longitudinal relationship between UA and CAC. Moreover, this study could not confirm that UA is associated with cardiovascular events in patients with CKD. CAC is a complex process; therefore, there could be confounders that we could not consider. However, this study is the first, to the best of our knowledge, to elucidate that UA showed a different relationship with CAC according to CKD stages.

## Conclusions

The UA level was significantly associated with CAC in early CKD but not in advanced CKD. Longitudinal studies are required to confirm the association between UA and CAC and the effect of UA lowering therapy for CAC in patients with CKD.

## Supplementary Information

**Additional file 1: Supplementary table 1.** Baseline characteristics of participants according to CAC 0, > 0-100, > 100-400, > 400.

## Data Availability

The datasets used and analysed during the current study available from the corresponding author on reasonable request.

## Abbreviations

CVD: Cardiovascular disease
CKD: Chronic kidney disease
CAC: Coronary artery calcification
UA: Uric acid
KNOW-CKD: KoreaN Cohort Study for Outcome in Patients With Chronic Kidney Disease
BP: Blood pressure
eGFR: Estimated glomerular filtration rate
OR: Odds ratio
CI: Confidence interval
