# Supplementary material for: Serum uric acid is associated with coronary artery calcification in early chronic kidney disease: a cross-sectional study
Source: BMC Nephrol. 2021 Jul 4;22:247. doi: 10.1186/s12882-021-02463-2 (PMC8255010; doi:10.1186/s12882-021-02463-2)
Supplement: Supplementary file 1 — Additional file 1: Supplementary table 1. Baseline characteristics of participants according to CAC 0, > 0-100, > 100-400, > 400. [file 12882_2021_2463_MOESM1_ESM.docx]

Supplementary table 1. Baseline characteristics of participants according to CAC 0, > 0-100, > 100-400, > 400

|  | Total Agatston score | | | | p-value | p-for trend |
| --- | --- | --- | --- | --- | --- | --- |
|  | CAC 0 | CAC >0-100 | CAC >100-400 | CAC >400 |  |  |
|  | (n = 645) | (n = 384) | (n = 150) | (n = 171) |  |  |
| Age, year | 47.6 (11.3) | 56.4 (10.0) | 60.0 (9.6) | 63.4 (7.5) | <.0001 | <0.001 |
| Sex, male (%) | 277 (43.0) | 240 (62.5) | 111 (74.0) | 130 (76.0) | <.0001 | <0.001 |
| current smoker (%) | 88 (13.6) | 67 (17.5) | 34 (22.7) | 28 (16.4) | 0.042 | 0.058 |
| Coronary artery disease (%) | 2 (0.3) | 16 (4.2) | 10 (6.7) | 42 (24.6) | <.0001 | <0.001 |
| Diabetes mellitus (%) | 97 (15.0) | 169 (44.0) | 96 (64.0) | 137 (80.1) | <.0001 | <0.001 |
| Hypertension (%) | 593 (91.9) | 377 (98.2) | 149 (99.3) | 167 (97.7) | <.0001 | <0.001 |
| Use of lipid-lowering drugs (%) | 279 (43.3) | 222 (57.8) | 104 (69.3) | 122 (71.3) | <.0001 | <0.001 |
| Mean blood pressure, mmHg | 93.1 (11.3) | 94.9 (10.9) | 94.8 (10.8) | 94.6 (14.5) | 0.049 | 0.016 |
| Waist hip ratio (%) | 0.88 (0.08) | 0.91 (0.06) | 0.91 (0.06) | 0.93 (0.06) | <.0001 | <0.001 |
| Creatinine, mg/dL | 1.44 (0.93) | 1.84 (1.15) | 2.17 (1.29) | 2.15 (1.22) | <.0001 | <0.001 |
| eGFR, ml/min/1.73m^2^ | 67.3 (33.9) | 51.6 (29.1) | 42.8 (26.4) | 40.6 (22.6) | <.0001 | <0.001 |
| Uric acid, mg/dL | 6.7 (2.0) | 7.1 (1.9) | 7.5 (1.8) | 7.5 (1.8) | <.0001 | <0.001 |
| Calcium, mg/dL | 9.2 (0.5) | 9.1 (0.6) | 9.1 (0.6) | 9.0 (0.6) | <.0001 | <0.001 |
| Phosphate, mg/dL | 3.6 (0.6) | 3.7 (0.7) | 3.8 (0.7) | 3.9 (0.8) | <.0001 | <0.001 |
| LDL cholesterol, mmol/L | 102.6 (29.9) | 97.8 (33.7) | 92.2(31.7) | 89.1 (31.9) | <.0001 | <0.001 |
| HDL cholesterol, mmol/L | 53.6 (15.5) | 47.9 (15.6) | 46.5 (16.0) | 44.3 (14.1) | <.0001 | <0.001 |
| Ln PTH, pg/mL | 3.85 (0.70) | 4.00 (0.79) | 4.10 (0.75) | 4.11 (0.73) | <.0001 | <0.001 |
| Ln 24hour urine protein, mg | 5.7 (1.9) | 6.4 (1.6) | 6.6 (1.6) | 6.7 (1.6) | <.0001 | <0.001 |

eGFR, estimated glomerular filtration rate; LDL, low density lipoprotein; HDL, high density lipoprotein; PTH, parathyroid hormone
